# Turning Diamagnetic Microbes into Multinary Micro-Magnets: Magnetophoresis and Spatio-Temporal Manipulation of Individual Living Cells

**DOI:** 10.1038/srep38517

**Published:** 2016-12-05

**Authors:** Hojae Lee, Daewha Hong, Hyeoncheol Cho, Ji Yup Kim, Ji Hun Park, Sang Hee Lee, Ho Min Kim, Rawil F. Fakhrullin, Insung S. Choi

**Affiliations:** 1 Center for Cell-Encapsulation Research, Department of Chemistry, KAIST, Daejeon 34141, Korea; 2 Graduate School of Medical Science and Engineering, KAIST, Daejeon 34141, Korea; 3 Bionanotechnology Lab, Institute of Fundamental Medicine & Biology, Kazan Federal University, Kreml uramı 18, Kazan, Republic of Tatarstan 420008, Russian Federation

## Abstract

Inspired by the biogenic magnetism found in certain organisms, such as magnetotactic bacteria, magnetic nanomaterials have been integrated into living cells for bioorthogonal, magnetic manipulation of the cells. However, magnetized cells have so far been reported to be only binary system (on/off) without any control of magnetization degree, limiting their applications typically to the simple accumulation or separation of cells as a whole. In this work, the magnetization degree is tightly controlled, leading to the generation of multiple subgroups of the magnetized cells, and each subgroup is manipulated independently from the other subgroups in the pool of heterogeneous cell-mixtures. This work will provide a strategic approach to tailor-made fabrication of magnetically functionalized living cells as micro-magnets, and open new vistas in biotechnological and biomedical applications, which highly demand the spatio-temporal manipulation of living cells.

Biological entities are generally diamagnetic, but some organisms, such as ants, honeybees, homing pigeons, salmons, and certain bacteria, have developed a fascinating strategy of utilizing magnetism as a toolbox for their survival^1^,^2^,^3^,^4^. For example, the chains of magnetosomes — ferrimagnetic nanoparticles covered with phospholipid bilayers — in magnetotactic bacteria are used as a compass needle for their active swimming toward a growth-favoring microoxic zone^5^,^6^,^7^. Juvenile salmons, with no prior migratory experience, locate specific oceanic feeding habitats, which are far from their natal sites, with the combined information on magnetic intensity and inclination angle from magnetic particles in their skull^8^. These examples show how nature effectively links the bioorthogonal properties of magnetism (*e.g.*, contactlessness, remoteness, and permeability) with biological processes and behavior for survival.

During the past decades, the bioorthogonality of magnetism has inspired researchers to integrate (super)paramagnetic nanomaterials into living cells and to generate magnetically functionalized hybrid cells, because spatio-selective manipulation of cells (*e.g.*, movement and rotation), without deteriorating biological activities, will be achieved with ease by external magnetic field^9^. The applications of magnetized cells include functional biomachines, such as micromotors and propellers^10^,^11^, as well as biosorbents and biocatalysts that are localized and concentrated magnetically in the device^12^. In addition, the magnetism also could be coupled with biological processes and control them in a programmed fashion^13^,^14^,^15^,^16^,^17^, reminiscent of the events found in nature aforementioned. For example, citrate-stabilized iron oxide magnetic nanoparticles (MNPs) were loaded into red blood cells (RBCs), and the movement of the ultrasound-powered RBCs was magnetically guided in undiluted whole blood, suggesting a biocompatible cargo delivery system *in vivo*^11^. The movement of neuron-like PC12 cells and their neurite orientation were manipulated in a controlled manner, when the external magnetic field was applied to MNP-internalized PC12 cells^13^,^14^. In addition to the endocytic internalization of MNPs into cells, direct deposition of MNPs [especially, poly(allylamine hydrochloride) (PAH)-stabilized MNPs] onto cell surfaces has been suggested, by us and others, as a powerful alternative for magnetization and applications of diamagnetic cells^18^,^19^,^20^,^21^. Compared with the internalization method, this process of deposition was simple and fast (in several minutes), and proved cytocompatible: microbial and mammalian cells have successfully been coated with PAH-stabilized MNPs without noticeable loss of cell viability and metabolic activities. Besides applications in biosensors and microfluidics, magnetized human cells were assembled into scaffold-free, 3D multicellular spheroids as a model of tissues^22^,^23^,^24^.

Albeit recent advances in cell magnetization (transformation of diamagnetic cells into (super)paramagnetic hybrids), the demonstrations have so far typically been limited to the simple accumulation or separation of cells as a whole. However, it is highly demanded, especially for microfluidic and *in vivo* applications^25^,^26^,^27^,^28^,^29^,^30^, that a specific subgroup of cells be manipulated, in the pool of heterogeneous cell-mixtures, independently from the other subgroups. This type of controlled cell manipulation, in principle, could be achieved by alternating external magnetic field or modulating the magnetic moment of magnetized cells; however, the synthetic challenge remains how to control the cell magnetization (*e.g.*, control over the surface density of MNPs and magnetic susceptibility). In other words, the cell magnetization has so far been limited to binary systems (yes and no from magnetized and non-magnetized cells), but “multinary” magnetization of living cells would allow for multi-level control of magnetized cells. We also envisioned that the multinary incorporation of magnetic nanomaterials into artificial spores (cell-in-shell structures)^31^,^32^,^33^,^34^,^35^ would provide an advanced tool for manipulating the cellular hybrid structures at the single-cell level. In this work, we developed a simple but versatile method for varying the magnetization degrees of individual living cells, as micro-magnets, while maintaining the cell viability. Specifically, the integration dimension of MNPs into cells was controlled by the layer-by-layer (LbL) process of MNP deposition and *in situ* bioinspired silicification (Fig. 1a). The superconducting quantum interference device (SQUID) magnetometric analysis showed that the magnetic susceptibility and saturation magnetization (*M*_S_) of the resulting magnetized cells were proportional to the integration degree of MNPs on individual cells. In addition, the anisotropic distribution of MNPs on cell surfaces provided a net magnetization, which enabled spatio-temporally selective magnetic alignment and magnetophoresis of living cells.

## Results

### Formation and Characterization of MSi on gold and yeast

**Figure 1:**
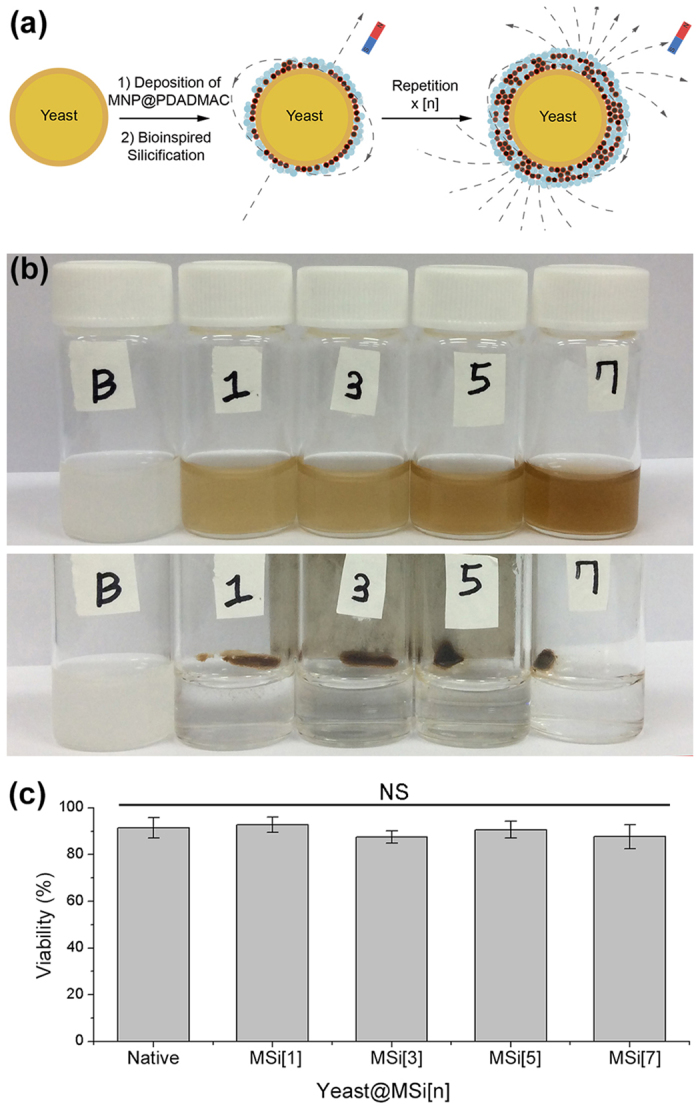
(**a**) Schematic representation for fabrication of yeast@MSi[n] based on the reactive LbL protocol composed of MNP deposition and *in situ* bioinspired silicification. The cycle of MNP deposition and silicification was repeated up to 7 times to vary the magnetization degrees. (**b**) Photographs of native yeast and yeast@MSi[n] (n = 1, 3, 5, and 7; B indicates native bare yeast cells): (top) before and (bottom) after magnetic attraction. (**c**) Viabilities of native yeast and yeast@MSi[n]. The viability was calculated based on the FDA assay. Independent experimental sets (N = 3) were used for analysis, and more than 700 cells were measured for each set. The error bars show the standard deviation (SD). The *p*-value of Kruskal-Wallis test (One-way ANOVA) was 0.2. NS: not significant.

The multinary magnetization of cells was primarily based on the thickness-controllable LbL strategy, where we applied a method of *in situ* bioinspired silicification to firmly incorporate MNPs into the LbL layer. We have previously reported that certain polyamines, such as poly(diallyldimethylammonium chloride) (PDADMAC) and poly(ethyleneimine) (PEI), catalyzed the polycondensation of silicic acid derivatives, leading to the formation of siliceous films, under physiologically relevant conditions^36^,^37^, and applied this bioinspired protocol to the silica nanocoating of individual cells for the fabrication of artificial spores^38^,^39^,^40^,^41^. We, based on the previous work, synthesized PDADMAC-stabilized MNPs (MNP@PDADMACs)^42^ and utilized them as a catalytically active (for silicification) and magnetic component in the LbL process (for characterization data, see Figs S1, S2 and S3). The catalytic activity of MNP@PDADMAC for *in situ* silicification was confirmed with a flat gold substrate as a model. After formation of carboxylate (COO^−^)-terminated self-assembled monolayers (SAMs) of 11-mercaptoundecanoic acid, mimicking negatively charged cell surfaces, the SAM-coated gold substrate was incubated alternatively in an aqueous NaCl solution of MNP@PDADMAC and a silicic acid derivative solution at room temperature. The MNP-deposition/silicification step (cycle) was repeated up to 7 times, leading to the formation of magnetic silica (MSi) films with different thicknesses (in other words, different numbers of MNPs). The silica formation was confirmed by grazing-angle Fourier-transform infrared (GA-FTIR) spectroscopy: the IR spectrum, after 7 cycles, showed the peaks at 1219, 974, and 800 cm^−1^, corresponding to Si-O-Si asymmetric stretching, Si-O^−^ stretching, and Si-O-Si symmetry stretching, respectively (Fig. S4). After confirming the formation of the MSi films, the synthetic protocol developed was applied to living *Saccharomyces cerevisiae* (baker’s yeast). Yeast cells (OD_600_ = 1.1, optical density at 600 nm) were incubated in an aqueous NaCl solution of MNP@PDADMAC for 5 min, and, after washing with a phosphate-buffered solution (pH 5.8), immersed for 10 min in an aqueous solution of silicic acid derivatives (see the Experimental Section for details). The cycle was repeated up to 7 times, generating yeast@MSi[n] (n = 1–7; number of cycles). The color of yeast@MSi[n] suspension became darker as the number of LbL cycles (n) increased, indicating different degrees of cell magnetization (Fig. 1b; for zeta potential data, see Fig. S5). The different magnetization degrees were also supported by the scanning electron microscopy (SEM) and transmission electron microscopy (TEM) images (Figs S6 and S7). The synthetic protocol employed was astonishingly cytocompatible: the fluorescein diacetate (FDA) assay, which assesses the esterase activity in metabolically intact cells, showed that the cell viability remained undiminished even after 7 cycles (Fig. 1c). Yeast@MSi[n] existed as an individual cell, not as a cell cluster that had chronically been observed in previous magnetized cells^18^,^19^,^20^.

### Controlled magnetization of yeast@MSi

**Figure 2:**
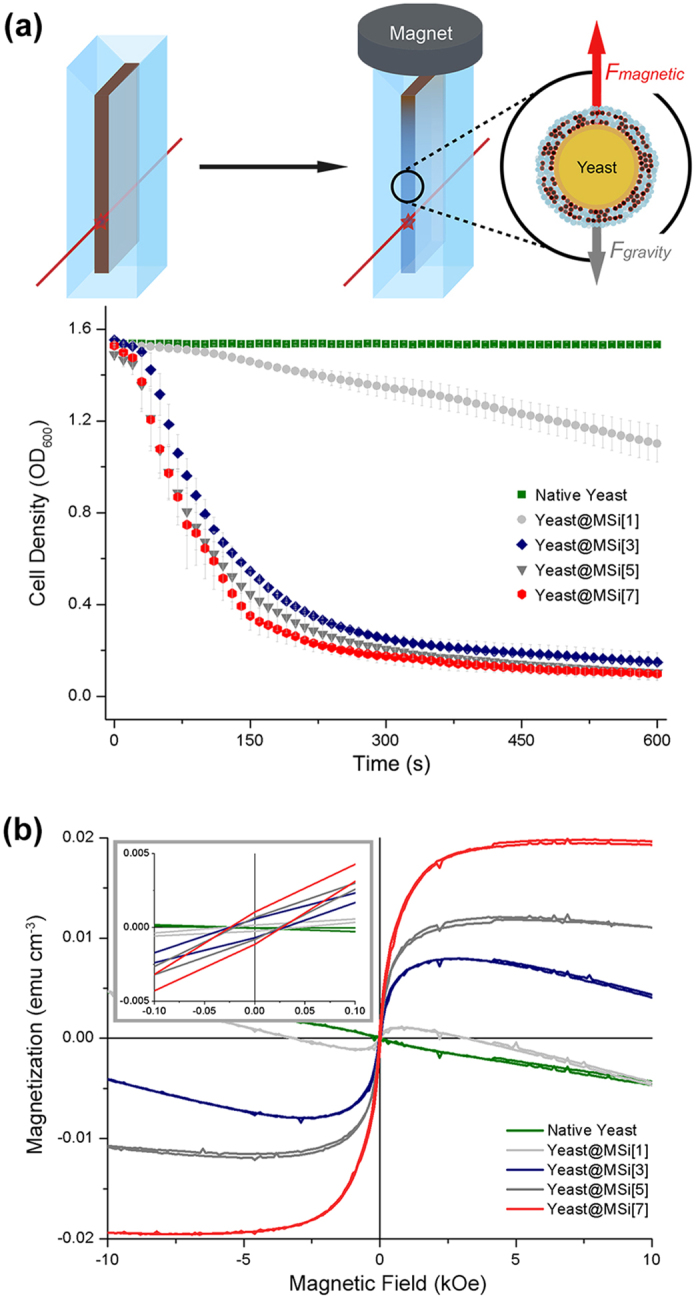
(**a**) Experimental setup for magnetic movement of cells and optical measurement. A round-shaped neodymium magnet (320 mT) was used. The optical density (OD) was measured at the wavelength of 600 nm. Graphs of OD_600_ vs. time of magnetic exposure for native yeast and yeast@MSi[n] (n = 1, 3, 5, and 7) under a permanent magnet. Independent experimental sets (N = 3) were used for statistical analysis, and the error bars show the standard deviation (SD). (**b**) Magnetic hysteresis curves of native and yeast@MSi[n] (n = 1, 3, 5, and 7) in the liquid state at 300 K by SQUID magnetometry.

The multinary behavior of yeast@MSi[n] (n = 1, 3, 5, and 7) was investigated with native yeast as a reference. Each set of cells was dispersed in a UV-Visible cuvette, on the top of which a permanent neodymium magnet was placed, and the OD_600_ values were measured every 10 sec with a UV-Visible spectrophotometer (Fig. 2a). In this setting, the magnetized yeast@MSi[n] was subject to the gradient of magnetic field and was attracted toward the magnet because of the magnetic dipole of MNPs. Therefore, the upward movement of the cells, against to the Earth’s gravity, decreased the measured OD_600_ value. The UV-Visible measurements confirmed that each set of yeast cells was magnetized to different degree: the OD_600_ values were 1.54 for native yeast, and 1.46, 0.55, 0.44, and 0.35 for yeast@MSi[n] (n = 1, 3, 5, and 7) at 150 sec of magnetic exposure. We also used SQUID magnetometry to acquire more quantitative data on multinary magnetization. The hysteresis curves were obtained in the liquid state at 300 K, and the graphs of the magnetization (*M*, magnetic dipole moment per unit volume) versus the applied magnetic field (*H*) were plotted (Fig. 2b). Figure 2b showed that the diamagnetic yeast cells became superparamagnetic after MSi coating: the *M* value increased with the increase of *H* and saturated at higher *H*, and the coercive field and remanent magnetization were negligible (inset of Fig. 2b). More importantly, the value of saturation magnetization (*M*_S_) was different from one another for yeast@MSi[n]: the values were 0.0012, 0.0080, 0.0123, and 0.0200 emu cm^−3^ for n = 1, 3, 5, and 7, respectively. Even in weak magnetic field of 10 mT (*i.e.*, 100 Oe)^14^,^15^,^21^, yeast@MSi[n] showed conspicuous differences in the magnetization (0.0006, 0.0024, 0.0032, and 0.0043 emu cm^−3^ for n = 1, 3, 5, and 7) and the corresponding magnetic susceptibility (1.08 × 10^−6^, 4.50 × 10^−6^, 6.02 × 10^−6^, and 8.07 × 10^−6^).

### Magnetophoresis of yeast@MSi

**Figure 3:**
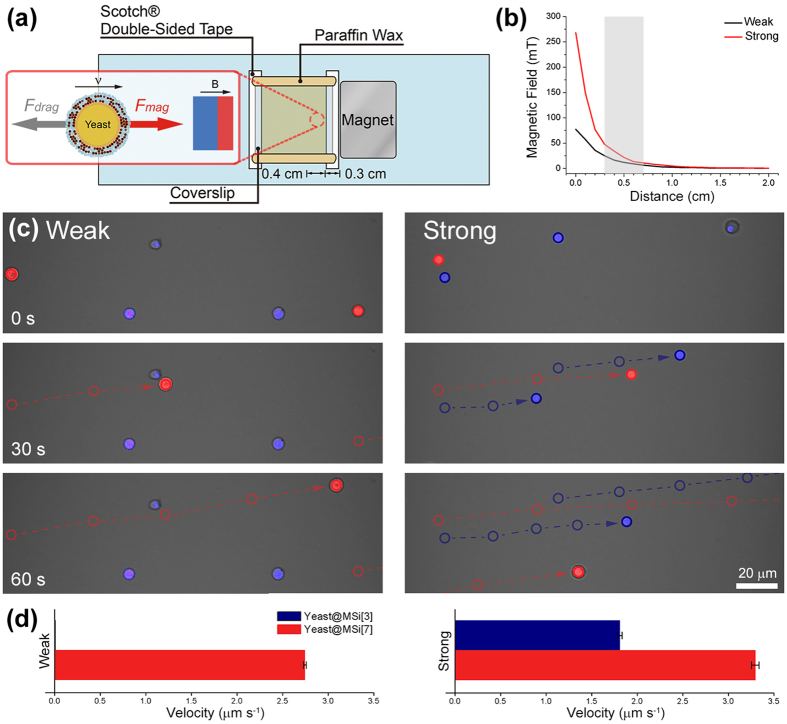
(**a**) Experimental setup for studies on the magnetophoresis of yeast@MSi. Inset: schematic representation of force balance between magnetic force and drag force, when yeast@MSi[n] moved with constant terminal velocity *v*. (**b**) Graph of the magnetic-field gradient generated by a magnet. The magnetic fields were measured on the 0.1-cm interval with a Gauss meter. Two types of permanent magnets (270 and 110 mT) were used for generating strong and weak magnetic fields, respectively. The movement of yeast@MSi[n] was investigated in the range of 0.3 to 0.7 cm from a permanent magnet. The working window was shaded in gray box. (**c**) Time-lapse CLSM images of yeast@MSi[3] (blue) and yeast@MSi[7] (red) under (left) weak and (right) strong gradients of the magnetic field. The images were taken every 3 sec, and the images at 0, 30, and 60 sec were presented. The cell movement with 15-sec interval was indicated with open circles and arrows. (**d**) Graph of velocity of yeast@MSi under the prepared magnetic-field gradient. The error bars show the standard deviation (SD). (N = 3).

To visualize the multinary magnetization, magnetophoresis was studied. Magnetophoresis is a kind of magnetic separation methods, where the velocity and trajectory of magnetized cells are determined by their magnetization degree and the strength of external magnetic-field gradient^43^. We took the time-lapse confocal laser-scanning microscopy (CLSM) images of yeast@MSi[3] and yeast@MSi[7] every 3 sec under external magnetic field and calculated the averaged velocity with triplicated experimental sets (Fig. 3). Yeast@MSi[3] and yeast@MSi[7] were stained with CellTraker^TM^ blue and red, respectively, and two types of permanent magnets were used for generating weak (ca. 46 mT cm^−1^, measured at the point of the working window) and strong (ca. 91 mT cm^−1^, measured at the point of the working window) magnetic-field gradients (Fig. 3a and b). As seen in the left panel of Fig. 3c, only yeast@MSi[7] (red) moved toward a magnet under weak magnetic field (to the right in the images). In contrast, the spatial location of yeast@MSi[3] (blue) was not changed under the conditions, although this subgroup of yeast cells was also magnetized. The movement velocity of yeast@MSi[7] was calculated to be 2.74 μm s^−1^, from the time-lapse images, in the weak magnetic field (Fig. 3d). The multinary magnetization was further confirmed by using a strong magnet. Under the strong magnetic field, both subgroups of cells moved to the right, but with different velocities (right panel in Fig. 3c). Yeast@MSi[7] moved faster than it did under weak field (velocity: 3.30 μm s^−1^), and the movement of yeast@MSi[3] was much slower, compared with that of yeast@MSi[7], with averaged velocity of 1.81 μm s^−1^ (Fig. 3d). Based on the measured velocity, we roughly estimated the number of MNPs per cell (*N*^*MNP*^) to be 2.71 × 10^4^ and 4.95 × 10^4^ for n = 3 and 7, respectively, by Equation (1) that assumed that the magnetic force acting on yeast@MSi[n], *F*_*mag*_, was balanced by the drag force, *F*_*drag*_ (for the parameters used for the calculations, see Table S1)^44^. Based on Stokes’ drag, we assumed the yeast@MSi[n] cell as a solid sphere and the MNP cluster on the cell surface as a single micro-sized magnetic particle^45^. When two forces were balanced, the yeast@MSi[n] cells reached their terminal velocity and showed constant-velocity movement (without acceleration). We also confirmed that the movement of yeast@MSi[n] was caused by the permanent magnet: changing the location of the magnet to the left made the cell movement changed directionally to the left (data not shown).


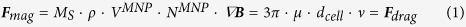


where *M*_S_ is the saturation magnetization of MNPs, *ρ* is the particle density, *V*^*MNP*^ is the volume of a MNP, *N*^*MNP*^ is the number of MNPs per cell, 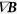 is the magnetic-field gradient, *μ* is the viscosity of water, *d*_*cell*_ is the cell diameter, and *ν* is the terminal velocity of cell.

### Magnetic alignment of yeast@MSi

**Figure 4:**
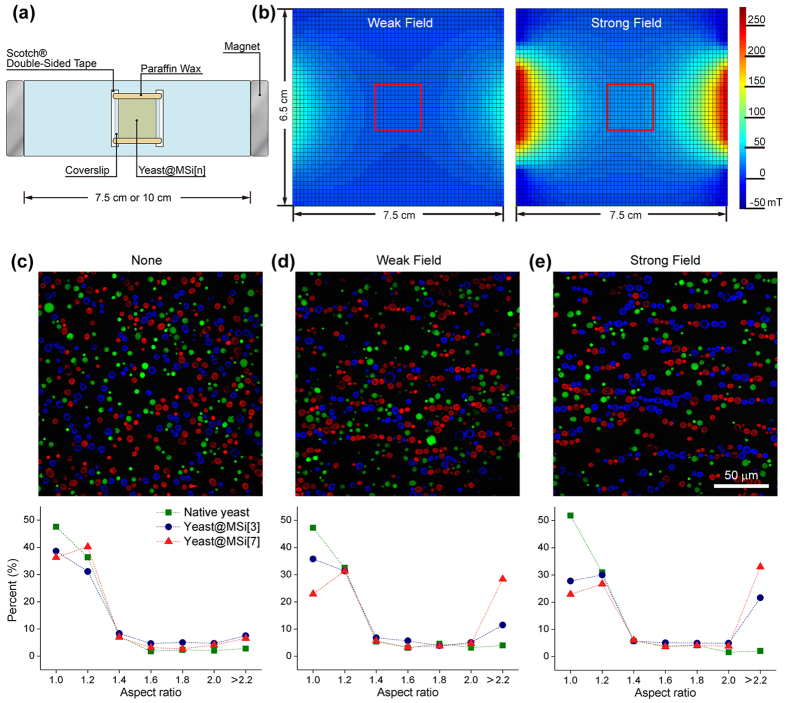
(**a**) Experimental setup for studies on 1D alignment of yeast cells. The strength of the magnetic field was controlled (weak or strong) by varying the distance between two permanent magnets (320 mT). Distance between two magnets: 7.5 cm for strong field and 10 cm for weak field. A yeast@MSi[n]-loaded slide glass (2.5 cm × 7.5 cm) was placed in the horizontal manner at the center of the magnetic field. (**b**) Magnetic-field gradients used for 1D alignment of yeast@MSi[n]. Magnetic fields were measured on the 0.5-cm interval with a Gauss meter and visualized with interpolation by MATLAB^®^. The red, open squares indicate the working window, in which the chamber was located and the magnetic alignment was observed. (**c**–**e**) Magnetic alignment behaviors of a mixture composed of native yeast, yeast@MSi[3], and yeast@MSi[7]: (**c**) before and (**d** and **e**) after application of magnetic field. Green: native yeast; blue: yeast@MSi[3]; red: yeast@MSi[7]. The aspect ratio of the assembled structures was measured, and the percent frequency graphs of native yeast (green square), yeast@MSi[3] (blue circle), and yeast@MSi[7] (red triangle) were presented on the graphs (bin: 0.2). Independent experimental sets (N > 4) were used for statistical analysis.

**Figure 5:**
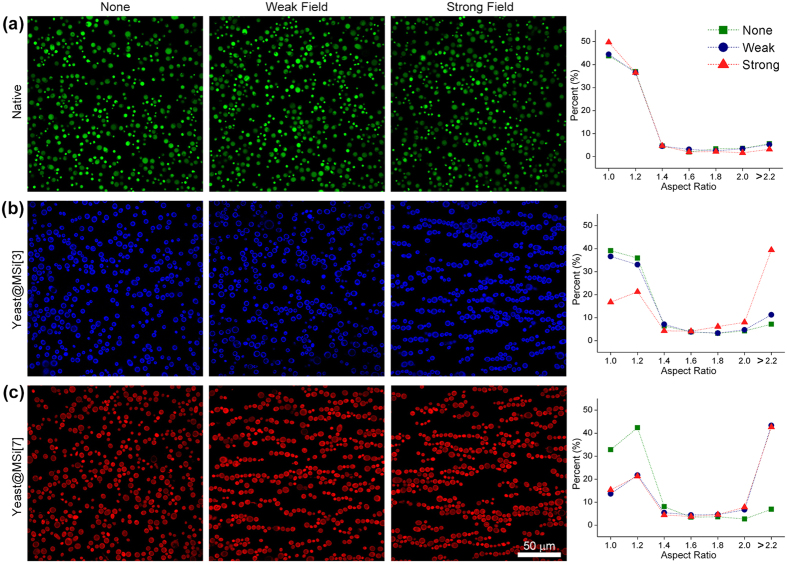
(**a**–**c**) Magnetic alignments of (**a**) native yeast (green), (**b**) yeast@MSi[3] (blue), and (**c**) yeast@MSi[7] (red). The right graphs show the percent frequencies of the aspect ratios of the assembled structures (bin: 0.2), clearly indicating the field-dependent alignment behavior of yeast@MSi[n]. Independent experimental sets (N > 4) were used for statistical analysis.

We demonstrated a proof-of-concept on the use of multinary magnetization for spatio-temporally selective one-dimensional (1D) alignment of cells (Fig. 4). To generate the pseudo-uniform magnetic field, permanent magnets were placed at both ends of a slide glass, and the distance between the two magnets was varied for modulating the field strength (strong or weak), as shown in Fig. 4a. The levels of the magnetic field were measured on the 0.5-cm interval with a Gauss meter, and the magnetic fields generated were visualized by MATLAB^®^, showing the strong (ca. 30 mT) and weak (ca. 13 mT) magnetic fields in the working window (Fig. 4b). To investigate the field-dependent behavior of yeast@MSi, we mixed native yeast (green), yeast@MSi[3] (blue), and yeast@MSi[7] (red), and placed the cell mixture under the magnetic field. The living yeast@MSi cells were observed to be highly responsive to the applied magnetic field and formed 1D lines (Fig. 4c–e; for images of individual channel, see Fig. S8). As expected, diamagnetic native yeast remained at the random locations under both weak and strong magnetic fields employed (Fig. 4c–e). In contrast, the 1D alignment behavior of yeast@MSi was observed to be field strength-dependent. The weak magnetic field enabled only yeast@MSi[7] to align themselves into 1D lines (Fig. 4d), but the increase in the magnetic field made both yeast@MSi[7] and yeast@MSi[3] self-assemble into 1D lines (Fig. 4e). The images in Fig. 4e also showed the heterogeneous 1D lines of yeast@MSi[n] (blue-and-red lines), allowing for compositional variations in 1D cellular architectures. The percent frequency graphs of the aspect ratio of the assembled structures (bin: 0.2) statistically showed the magnetization-dependent propensity for 1D alignment of yeast@MSi (Fig. 4c–e, bottom panel). The aspect ratio of single yeast cells was 1.0 to 1.4 values, because of their oval shape. After magnetic alignment, the aspect ratio of the 1D assembled structures increased in proportion to the number of aligned yeast cells with the greater than 2.0 value for dimeric assemblies. We also investigated magnetic alignment of each yeast@MSi[n] subgroup to acquire more detailed information on magnetization-dependent 1D alignment (Fig. 5). Consistent with the behavior of the cell mixture, each subgroups of yeast cells responded to and clearly formed 1D assemblies under applied magnetic field. The weak field made only yeast@MSi[7] aligned into 1D lines, while the strong field enabled the alignment of both yeast@MSi[3] and yeast@MSi[7]. Native yeast remained random under both magnetic fields. The self-assembly of magnetic materials is one of the most representative characteristics. The 1D-structured MNPs show maximum uniaxial anisotropy, which forces the direction of magnetization to lie along the linear structure, and have increased magnetic properties compared with individual MNPs. In our system, yeast@MSi[n] responded to the external magnetic field and aligned themselves to 1D lines, as bulk-sized magnetic particles did. The observation implied that yeast@MSi[n] were well magnetized and acted as single paramagnetic particles. Under external magnetic field, individual yeast@MSi[n] would be magnetized, making its own magnetic field (Fig. 1a); if the induced magnetic field around yeast@MSi[n] cells was strong enough to make the cells close to each other, the yeast@MSi[n] cells would be aligned into 1D lines for maximizing uniaxial anisotropy either homogenously (Fig. 4) or heterogeneously (Fig. 5).

## Discussion

The main purpose of cell encapsulation (*i.e.*, formation of “cell-in-shell” structures) is to temporarily protect the cells against external stress during manipulation and storage, and to endow the cells with exogenous properties, magnetism in this work^31^,^32^,^33^,^34^. Living cells are generally vulnerable to chemical materials and treatments, and, therefore coating materials and procedures should be cytocompatible^35^,^36^,^37^,^38^,^39^,^40^,^41^. In this work, we chose polyamine-stabilized MNPs and silica as coating materials, because these materials proved highly cytocompatible, even in the case of mammalian cells (*e.g.*, HeLa cells), in our previous studies^18^,^39^. After cell encapsulation with the MSi shells, the cell viability was maintained without noticeable decrease, and yeast@MSi retained their basic metabolism and functions, such as cell division, because small molecules, such as O_2_ and nutrients, could pass through the MSi shells^35^. The UV-Visible and SQUID analyses confirmed that the multinary magnetization of yeast cells was achieved by the reactive LbL method that controlled the number of MNPs deposited onto the cell wall. As shown in the SEM and TEM images (Figs S6 and S7), the original oval shape of yeast cells was maintained regardless of the deposition number of layers [n]. Consistent with the UV-Visible and SQUID analyses, the MSi shell was not observed clearly in the SEM and TEM images of yeast@MSi[1]. The nanoparticulate structures of MSi were seen for yeast@MSi[3], and the surfaces became rougher with the number of deposition layers [n].

The net magnetization, from anisotropic distribution of MNPs on cell surfaces, enabled magnetophoresis and magnetic alignment of yeast@MSi[n]. The magnetophoresis study showed that each and every subgroup of the magnetized cells could be separated and manipulated independently from one another by modulating the external magnetic field. The multinary magnetization of living cells, therefore, provided additional factor that directly enhanced the resolution of magnetophoresis. In typical magnetophoresis, the velocity and trajectory of magnetized cells are simply decided by cell diameter and field strength, limiting the use of magnetophoresis to the simple yes/no binary separation (from magnetized and non-magnetized cells). Our method would facilitate advanced magnetophoresis, which allows for multinary separation by alternating external magnetic field, and be applied to microfluidic-based biosensors^46^. In addition, multiple subgroups of yeast@MSi[n] would be utilized as a marker, like a protein-size marker for electrophoresis, for guiding unknown samples. Taken together, the results showed that the spatio-temporal control (*e.g.*, movement and assembly/disassembly) of each subgroup of the yeast@MSi cells could be achieved by modulation of magnetic fields. This characteristics of our system was differentiated from the natural system of magnetosomes, the assembly of which is template-driven, not magnetic field-controlled^47^, and would provide advanced chemical versatility in the manipulation of magnetized living cells.

In conclusion, this work demonstrated a tailor-made mimicry of the magnetized cells found in nature to some extent, with exogenous characteristics of multinary magnetization. A highly cytocompatible method was developed for fabricating various sets of magnetized cells as micro-magnets. Each subgroup of yeast@MSi behaved differently under external magnetic field in the aspects of movement velocity and 1D assembly. Although we used one type of cells, yeast cells, for the demonstration, the experimental handiness of the developed method would allow for advanced magnetophoresis and spatio-temporal magnetic manipulation of cells in the heterogeneous mixture of many different cell types including mammalian cells. We believe that the reversible homotypic or heterotypic assembly/disassembly, controlled by magnetic field, would contribute, especially, to studies on cellular functions, such as pathogenic invasion and cell-cell interactions. In addition, it is envisioned that the “multinary” system of magnetized living cells is combined with other techniques, such as magnetic tweezer, for single cell-level manipulation and analysis. The anisotropic magnetization of individual living cells, with multinary magnetization, will further advance the field of magnetized cells, which is our next research thrust.

## Methods

### Materials

Iron(II) chloride tetrahydrate (FeCl_2_∙4H_2_O, 99.0%, Aldrich), iron(III) chloride hexahydrate (FeCl_3_∙6H_2_O, 99.0%, Aldrich), sodium hydroxide (NaOH, Daejung), poly(diallyldimethylammonium chloride) (PDADMAC, MW: 100,000–200,000, 20 wt%, in H_2_O, Aldrich), tetramethyl orthosilicate (TMOS, 99%, Aldrich), sodium phosphate dibasic (Na_2_HPO_4_, 99%, Aldrich), sodium dihydrogen phosphate (NaH_2_PO_4_, 99%, Aldrich), sodium chloride (NaCl, 99%, Jin Chemical), 11-mercaptoundecanoic acid (MUA, 95%, Aldrich), sulfuric acid (H_2_SO_4_, 95%, Junsei), hydrogen peroxide (H_2_O_2_, 30–35%, Junsei), hydrochloric acid (HCl, 35%, Junsei), fluorescein diacetate (FDA, Aldrich), Cell Tracker^TM^ Green CMFDA (Invitrogen), Cell Tracker^TM^ Violet BMQC (Invitrogen), Cell Tracker^TM^ Orange CMRA (Invitrogen), yeast-extract-peptone-dextrose broth (YPD broth, Duchefa Biochemistry), yeast-extract-peptone-dextrose agar (YPD agar, Duchefa Biochemistry), absolute ethanol (99.8%, Merck), and acetone (99.8%, Merck) were used as received. Ultrapure water (18.3 MΩ∙cm) from the Human Ultrapure System (Human Corp.) was used. Permanent neodymium magnets (110 mT (15 × 10 × 1 mm), 270 mT (15 × 10 × 4 mm), and 320 mT (D30 × 10 mm), Umagnet) were used.

### Synthesis and Characterization of PDADMAC-Stabilized MNPs (MNP@PDADMACs)

Iron oxide MNPs were prepared by the co-precipitation method^35^. Briefly, 1.73 g of FeCl_3_∙6H_2_O and 0.635 g of FeCl_2_∙4H_2_O (molar ratio, Fe(III):Fe(II) = 2:1) were completely dissolved in 5 mL of distilled water, followed by adding 0.17 mL of 12-M HCl solution with stirring. The resulting yellowish solution was added dropwise to 50 mL of 1.5-M NaOH solution with vigorous stirring. The resulting dark magnetite was collected with a permanent magnet and washed with distilled water several times until the pH value of the supernatant reached ~9. The as-made MNPs were immersed into an aqueous PDADMAC solution (10 mg mL^−1^) and sonicated for 30 min. After sonication, MNP@PDADMACs were collected by centrifugation at 10,000 rpm for 15 min and washed with distilled water twice to remove the remaining free PDADMAC from the suspension. Distilled water, used in the synthesis, was deoxygenated by bubbling with argon gas for 6 h. The zeta potential and dynamic light scattering (DLS) analyses of MNP@PDADMACs were performed in an aqueous solution at 25 °C with Nano ZS90 (Malvern). Transmission electron microscopy (TEM) images were acquired by using a HEOL FB2100F instrument operated at 200 kV.

### MSi Formation on Gold

A gold substrate was cleaned for 10 min in piranha solution (v/v 70% H_2_SO_4_/30% H_2_O_2_), rinsed with distilled water, and dried with a flow of argon gas. The gold substrate was immersed in an ethanolic solution of MUA (1 mM) for 18 h to form the self-assembled monolayers (SAMs) terminating in negatively charged carboxylate. The SAM-coated substrate was incubated in a 150-mM aqueous NaCl solution of MNP@PDADMACs (0.1 mg mL^−1^) for 5 min, and, after washing with aqueous sodium phosphate-buffered (PB, 50 mM, pH 5.8) solution, immersed for 10 min in the 100-mM silicic acid derivative solution that had been prepared by stirring an HCl solution (1 mM) of TMOS (1 M) at room temperature for 20 min and adding the resulting solution to a PB solution with 1:9 (v/v) ratio. The cycle of MNP deposition and bioinspired silicification was repeated with the predetermined number from 1 to 7. The substrate was washed with a PB solution after each step. IR spectra were recorded with an FT-IR spectrometer (Thermo Nicolet Nexus).

### MSi Formation on Yeast

For fabrication of yeast@MSi[n], a single colony of yeast cells was picked from a YPD agar plate, suspended in the YPD broth, and cultured in a shaking incubator at 30 °C for 30 h. The cells were washed with a 150-mM aqueous NaCl solution twice. The procedures for MSi formation on yeast were the same as those for the gold substrate. Scanning electron microscopy (SEM) imaging was performed with an S-4800 field-emission scanning electron microscope (FE-SEM, Hitachi Co.) with an accelerating voltage of 15 kV after sputter-coating with platinum. TEM imaging of microtomed yeast cells was performed by using a Tecnai-G2 Spirit Twin instrument (FEI Co.). Zeta potential for yeast cells was measured using Nano ZS90 (Malvern) in deionized water at 25 °C.

### Viability Test

Cell viability was measured with FDA. FDA was dissolved in acetone (10 mg mL^−1^), and the 2 μL of the FDA stock solution was mixed with 1 mL of a yeast-cell suspension (PB, 50 mM, pH 6.5) for 30 min at 30 °C while shaking. The cells were collected by centrifugation, washed with a 150-mM aqueous NaCl solution, and characterized with a LSM 700 confocal microscope (Carl Zeiss).

### Magnetization of Yeast@MSi[n]: UV-Visible and SQUID Studies

The optical density of yeast cells (OD_600_) was measured at 600 nm with a UV-2550 UV-Visible spectrophotometer (Shimadzu). A 320-mT permanent neodymium magnet was used for the UV-Visible studies. Magnetic hysteresis curves were obtained with a SQUID magnetometer (Quantum Design MPMS) in the liquid state (15 μL). A customized polyether ether ketone (PEEK) tube was used for the liquid-state SQUID measurements.

### Magnetophoresis of Yeast@MSi[n]

Two strips of 0.3-cm-wide Scotch^®^ double-sided tape were attached on a slide glass 1.5 cm apart. A coverslip was placed on the tape supports to form a chamber, which was loaded with yeast@MSi[n]. The exposed edges were then sealed using paraffin wax. A permanent neodymium magnet (110 mT or 270 mT) was placed next to the chamber to generate the magnetic gradient field. The movement of yeast@MSi[n] was tracked by time-lapse imaging at every 3 sec with a LSM 700 confocal microscope (Carl Zeiss).

### Magnetic Alignment of Yeast@MSi[n]

The chamber, used for the magnetic-movement studies, was also utilized for magnetic alignment. Two permanent magnets (320 mT) were used for the magnetic-alignment studies. The strength of the magnetic field was controlled (weak or strong) by varying the distance between the two magnets. The distance between the magnets was set to be 7.5 cm for strong field or 10 cm for weak field. A yeast@MSi[n]-loaded chamber (2.5 cm × 7.5 cm) was placed in the horizontal manner at the center of the magnetic field. After 3-min exposure to the magnetic field, paraffin wax was used to sealing the chamber, and the magnetic alignment of yeast@MSi[n] was characterized with a LSM 700 confocal microscope (Carl Zeiss).

### Magnetic-Field Measurement

A digital Gauss meter, TM-801 (Kanetec), was used to measure the magnetic field induced by a permanent magnet.

## Additional Information

**How to cite this article**: Lee, H. *et al*. Turning Diamagnetic Microbes into Multinary Micro-Magnets: Magnetophoresis and Spatio-Temporal Manipulation of Individual Living Cells. *Sci. Rep.* **6**, 38517; doi: 10.1038/srep38517 (2016).

**Publisher's note:** Springer Nature remains neutral with regard to jurisdictional claims in published maps and institutional affiliations.

## Supplementary Material

Supplementary Information

Supplementary Movie S1

Supplementary Movie S2
