# Supplementary material for: Turning Diamagnetic Microbes into Multinary Micro-Magnets: Magnetophoresis and Spatio-Temporal Manipulation of Individual Living Cells
Source: Sci Rep. 2016 Dec 5;6:38517. doi: 10.1038/srep38517 (PMC5137033; doi:10.1038/srep38517)
Supplement: Supplementary Information [file srep38517-s1.pdf]

Supplementary Information

Turning Diamagnetic Microbes into Multinary Micro-Magnets: Magnetophoresis and Spatio-Temporal Manipulation of Individual Living Cells

Hojae Lee[1], Daewha Hong[1], Hyeoncheol Cho[1], Ji Yup Kim[1], Ji Hun Park[1], Sang Hee Lee[2], Ho Min Kim[2], Rawil F. Fakhrullin[3], and Insung S. Choi*[1]

[1] Center for Cell-Encapsulation Research, Department of Chemistry, KAIST, Daejeon 34141, Korea.

[2] Graduate School of Medical Science and Engineering, KAIST, Daejeon 34141, Korea.

[3] Bionanotechnology Lab, Institute of Fundamental Medicine & Biology, Kazan Federal University, Kreml uramı 18, Kazan, Republic of Tatarstan 420008, Russian Federation

*To whom correspondence should be addressed
E-mail: ischoi@kaist.ac.kr

CONTENTS

- **Table S1.** Parameters used in mathematical model.
- **Figure S1.** TEM micrographs of MNP@PDADMACs.
- **Figure S2**. Zeta potentials of MNPs and MNP@PDADMACs.
- **Figure S3.** Hydrodynamic diameters of MNPs and MNP@PDADMACs.
- **Figure S4.** GA-FTIR spectra of MNP@PDADMACs and MSi films on gold.
- **Figure S5.** Zeta potentials of yeast cells alternatively coated with MNPs and silica.
- **Figure S6.** SEM micrographs of native yeast and yeast@MSi[n].
- **Figure S7.** TEM micrographs of native yeast and yeast@MSi[n].
- **Figure S8.** Individual channel images of Figure 4.
- **Movie S1.** Magnetophoresis of yeast@MSi[3] and yeast@MSi[7] under weak field.
- **Movie S2.** Magnetophoresis of yeast@MSi[3] and yeast@MSi[7] under strong field.

Table S1. Parameters used in mathematical model.

Parameter	Descriptions	Value		Reference
M_S	M_S of MNPs	84.2 emu g^{-1} **		*
ρ	particle density	5 g cm^{-3}		(1)
V^{MNP}	volume of a MNP	7.30×10^{-25} m^3		*
∇B	magnetic field gradient	9.1 T m^{-1}		*
μ	viscosity of water	8.90×10^{-4} Pa·s		(2)
d_{Cell}	diameter of cell	5×10^{-6} m		*
v	terminal velocity of cell	n = 3	1.81×10^{-6} m s^{-1}	*
		n = 7	3.30×10^{-6} m s^{-1}	*
N^{MNP}	number of MNP per cell	$n = 3$	2.71×10^{4}	
		$n = 7$	4.95×10^{4}	

* obtained experimentally.

** 1 emu cm^{-3} = 10^3 A m^{-1}

(1) a) Riggio, C.; Calatayud, M. P.; Giannaccini, M.; Sanz, B.; Torres, T. E.; Femandez-Pacheco, R.; Ripoli, A.; Ibarra, M. R.; Dente, L.; Cuschieri, A.; Goya, G. F.; Raffa, V. The Orientation of the Neuronal Growth Process can be Directed via Magnetic Nanoparticles under an Applied Magnetic Field, *Nanomed. Nanotechnol. Biol. Med.* **10**, 1549-1558 (2014); b) Alon, N.; Havdala, T.; Skaat, H.; Raranes, K.; Marcus, M.; Levy, I.; Magel, S.; Sharoni, A.; Shefi, O. Magnetic Micro-Device for Manipulating PC12 Cell Migration and Oragnization. *Lab Chip* **15**, 2030-2036 (2015).

(2) Kestin, J.; Sokolov, M.; Wakeham, W. A. Viscosity of Liquid Water in the Range -8 °C to 150 °C, *J. Phys. Chem. Ref. Data* **7**, 941-948 (1978).

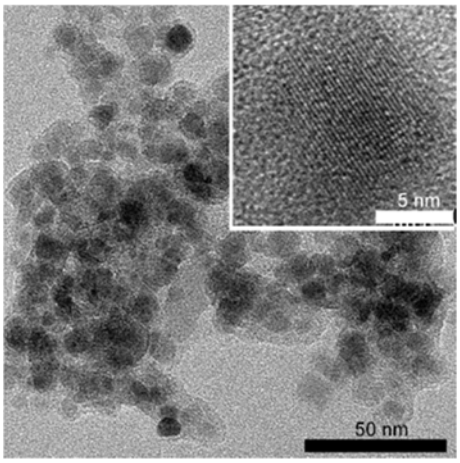

Figure S1. TEM micrographs of MNP@PDADMACs. The inset shows that MNPs are single-crystalline.

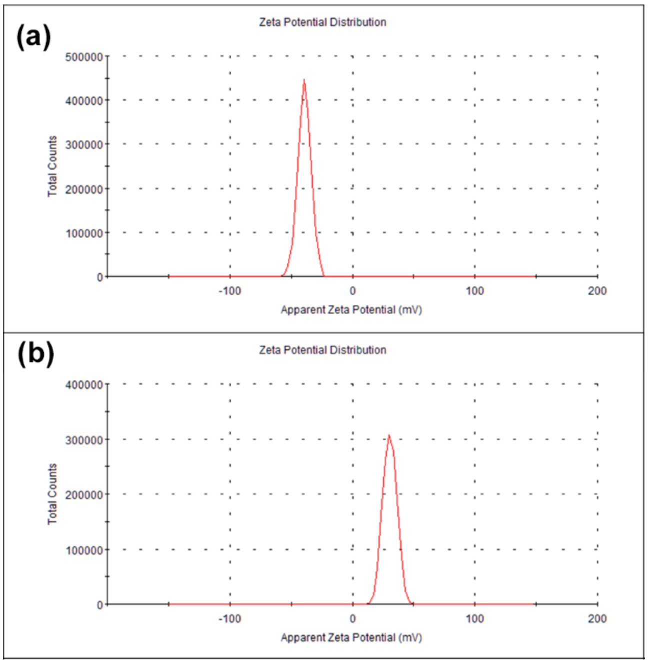

Figure S2. Zeta potentials of (a) MNPs and (b) MNP@PDADMACs. The PDADMAC-based stabilization of MNPs changed the zeta potential of the MNPs from negative (-39.2 ± 5.34 mV) to positive (30.5 ± 5.58 mV).

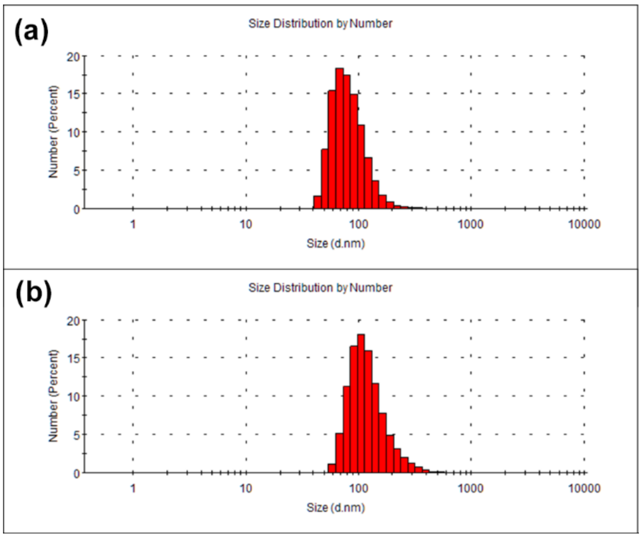

Figure S3. Hydrodynamic diameters of (a) MNPs and (b) MNP@PDADMACs by DLS measurements.

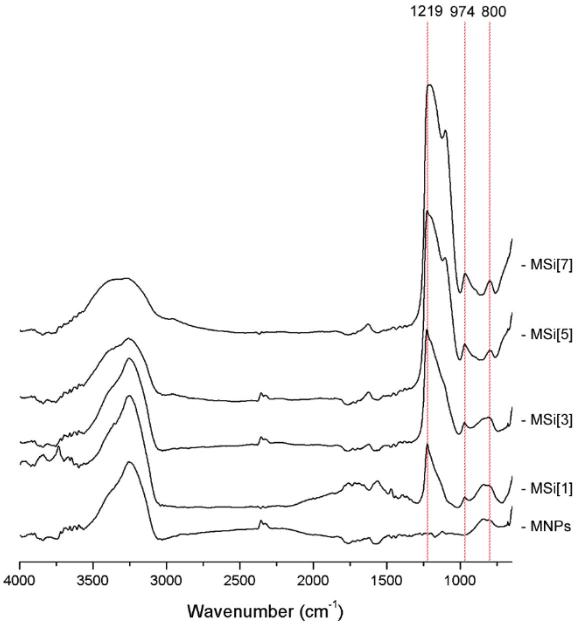

Figure S4. GA-FTIR spectra of MNP@PDADMACs and MSi films on gold. The IR peaks at 1219, 974, and 800 cm^{-1} correspond to Si-O-Si asymmetric stretching, $Si-O^-$ stretching, and Si-O-Si symmetry stretching, respectively.

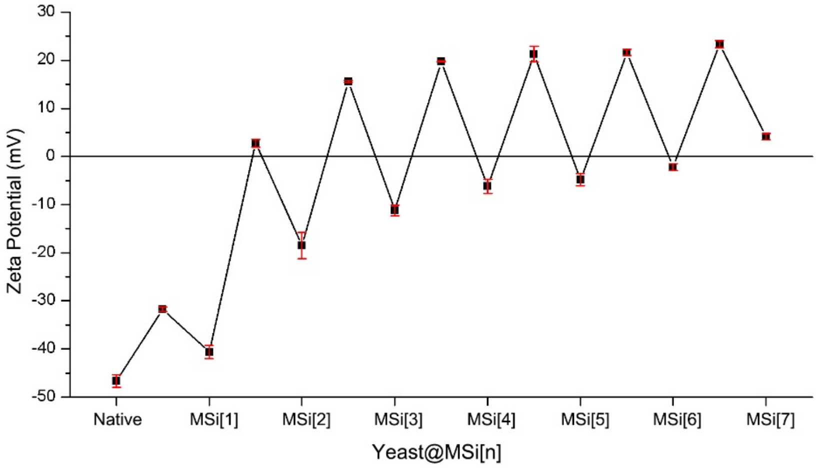

Figure S5. Zeta potentials of yeast cells alternatively coated with MNPs and silica. The zeta potential measurements show the periodic oscillation between positive and negative values after MSi[2] step, indicating successful deposition of MNP@PDADMACs and *in situ* silicification. Independent experimental sets (N>5) were used for statistical analysis.

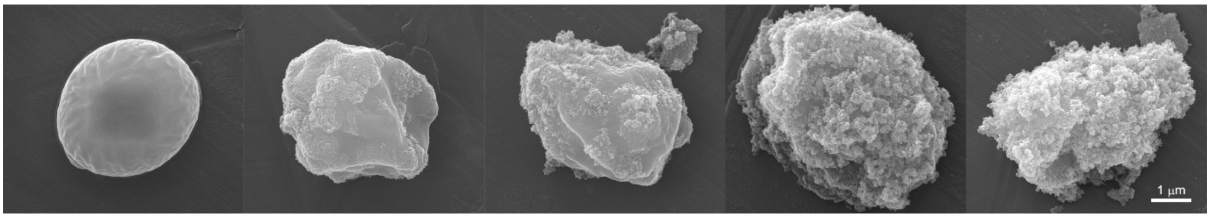

Figure S6. SEM micrographs of native yeast and yeast@MSi[n] (n= 1, 3, 5, and 7) from left.

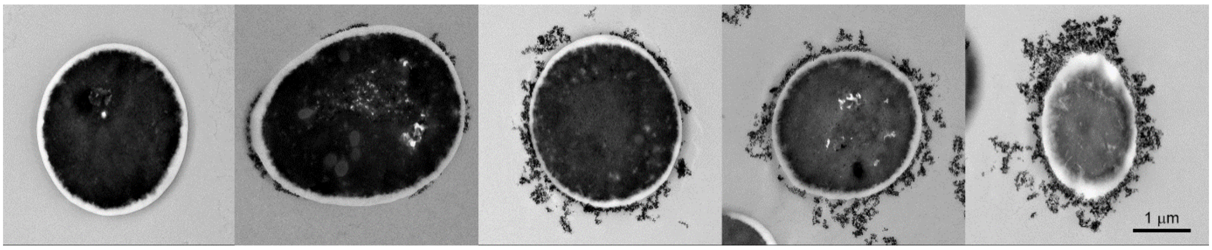

Figure S7. TEM micrographs of native yeast and yeast@MSi[n] (n= 1, 3, 5, and 7) from left.

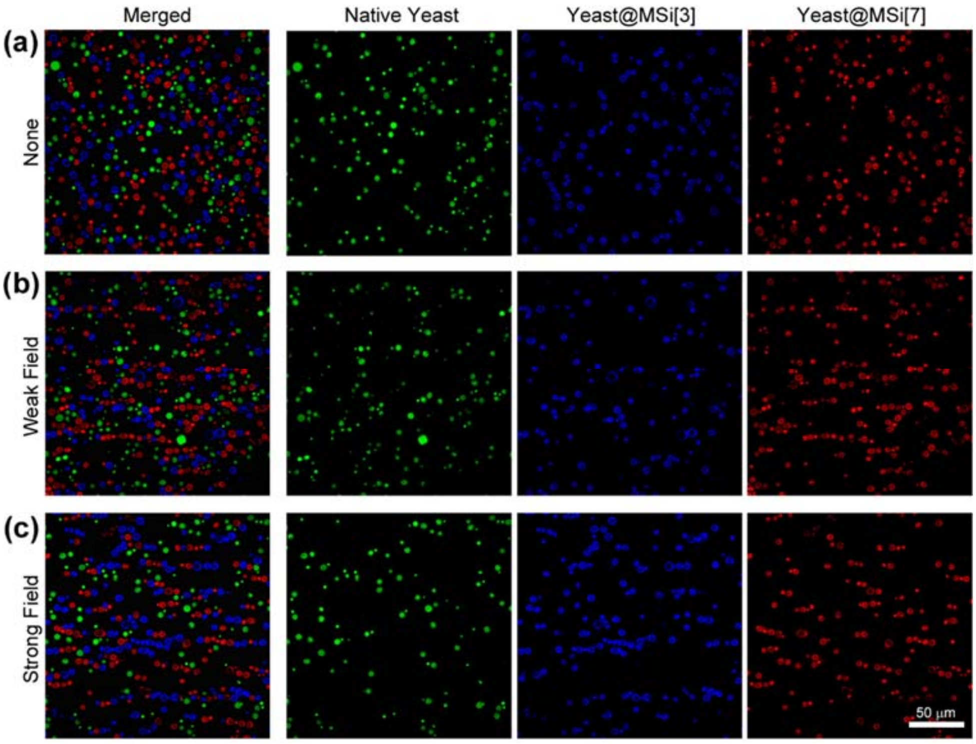

Figure S8. Individual channel images of Figure 4. Magnetic alignments of native yeast (green), yeast@MSi[3] (blue), and yeast@MSi[7] (red); (a) without magnetic field, (b) under weak, and (c) strong magnetic field.
